# Retraction Note: Desflurane anesthesia worsens emergence agitation in adult patients undergoing thyroid surgery compared to sevoflurane anesthesia

**DOI:** 10.1186/s40981-020-00320-z

**Published:** 2020-02-14

**Authors:** Takeshi Suzuki, Takuya Kurazumi, Tomomi Ueda, Hiromasa Nagata, Takashige Yamada, Shizuko Kosugi, Saori Hashiguchi, Koichi Ito, Hiroshi Morisaki

**Affiliations:** 1 Department of Anesthesiology, Keio University School of Medicine, 35 Shinanomachi, Shinjuku-ku, Tokyo, 160-8582 Japan; 2 Department of Surgery, Ito Hospital, Tokyo, Japan

**Correction to: JA Clin Rep (2017) 3:36**

**https://doi.org/10.1186/s40981-017-0106-5**

**Retraction**

The Editor-in-Chief has retracted this article [1]. The ethics committee approval was granted for an observational study and the need for patient consent was waived. However, the study design described is a randomized controlled trial and therefore patient consent should have been obtained. All authors agree with this retraction.
